# Involvement of Endoplasmic Reticulum Stress in TULP1 Induced Retinal Degeneration

**DOI:** 10.1371/journal.pone.0151806

**Published:** 2016-03-17

**Authors:** Glenn P. Lobo, Adrian Au, Philip D. Kiser, Stephanie A. Hagstrom

**Affiliations:** 1 Department of Ophthalmic Research, Cole Eye Institute, Cleveland Clinic, Cleveland, Ohio, 44195, United States of America; 2 Department of Pharmacology, Case Western Reserve University, Cleveland, Ohio, 44106, United States of America; 3 Louis Stokes Cleveland VA Medical Center, Cleveland, Ohio, 44106, United States of America; 4 Department of Ophthalmology, Cleveland Clinic Lerner College of Medicine of Case Western Reserve University, Cleveland, Ohio, 44195, United States of America; University of Florida, UNITED STATES

## Abstract

Inherited retinal disorders (IRDs) result in severe visual impairments in children and adults. A challenge in the field of retinal degenerations is identifying mechanisms of photoreceptor cell death related to specific genetic mutations. Mutations in the gene *TULP1* have been associated with two forms of IRDs, early-onset retinitis pigmentosa (RP) and Leber congenital amaurosis (LCA). TULP1 is a cytoplasmic, membrane-associated protein shown to be involved in transportation of newly synthesized proteins destined for the outer segment compartment of photoreceptor cells; however, how mutant TULP1 causes cell death is not understood. In this study, we provide evidence that common missense mutations in *TULP1* express as misfolded protein products that accumulate within the endoplasmic reticulum (ER) causing prolonged ER stress. In an effort to maintain protein homeostasis, photoreceptor cells then activate the unfolded protein response (UPR) complex. Our results indicate that the two major apoptotic arms of the UPR pathway, PERK and IRE1, are activated. Additionally, we show that retinas expressing mutant TULP1 significantly upregulate the expression of CHOP, a UPR signaling protein promoting apoptosis, and undergo photoreceptor cell death. Our study demonstrates that the ER-UPR, a known mechanism of apoptosis secondary to an overwhelming accumulation of misfolded protein, is involved in photoreceptor degeneration caused by missense mutations in *TULP1*. These observations suggest that modulating the UPR pathways might be a strategy for therapeutic intervention.

## Introduction

**Fig 1:**
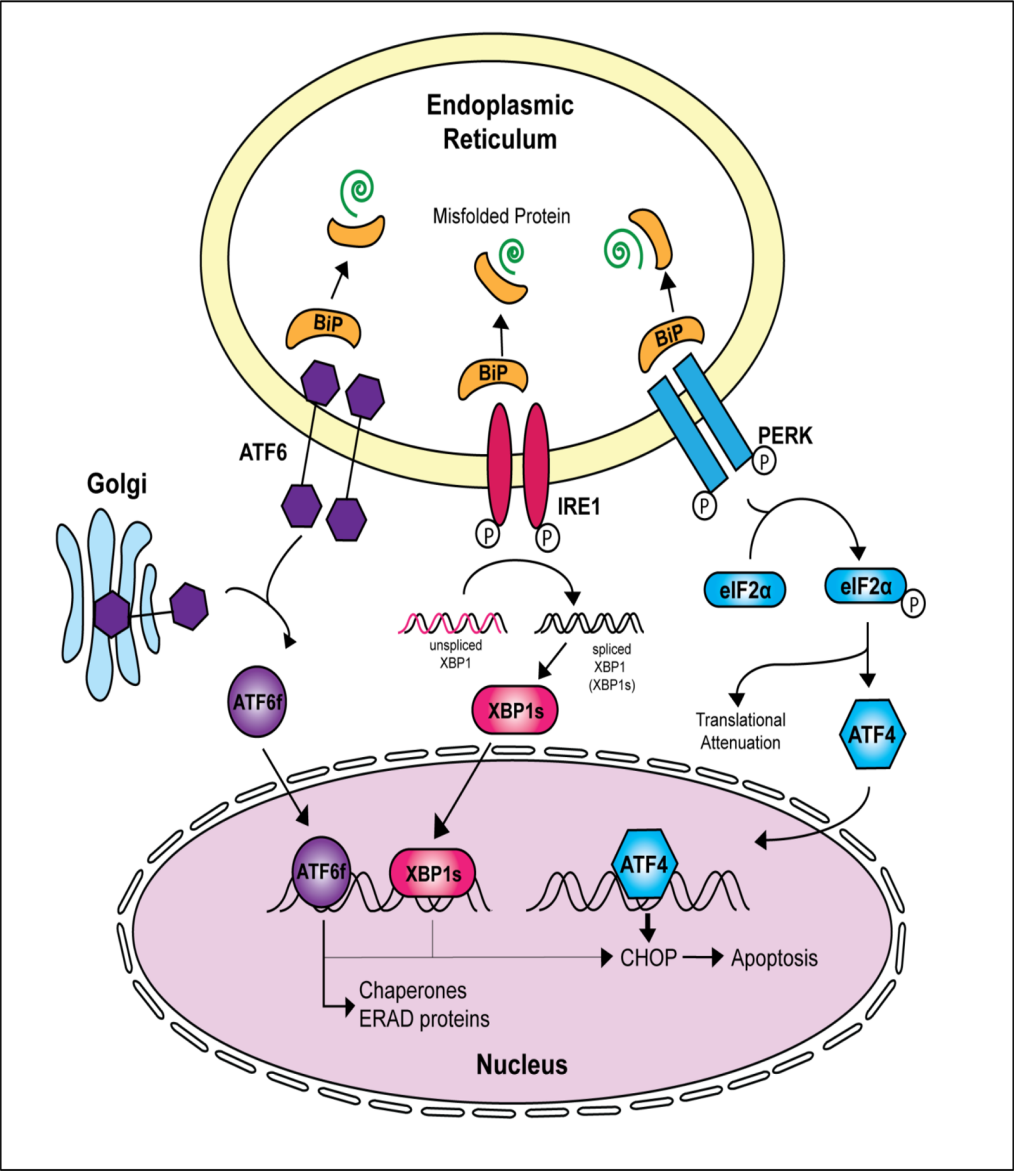
Activation of the Unfolded Protein Response (UPR) complex during misfolded protein accumulation within the ER. Accumulation of misfolded proteins in the ER lumen causes the chaperone protein BiP to dissociate from the three stress transducers PERK, IRE1 and ATF6. Loss of BiP binding, allows for either oligomerization or phosphorylation of the ER transducers, resulting in activation of their corresponding downstream signaling pathways. Prolonged signaling by the ER transducers and an inability of the cell to eliminate misfolded proteins via the endoplasmic reticulum associated degradation (ERAD) pathway leads to an upregulation of the pro-apoptotic transcription factor CHOP.

In humans, vision is paramount for quality of life and the impairment of sight represents a highly incapacitating condition. In retinal degenerative diseases, dysfunction or death of the rod and cone photoreceptor cells is the primary cause of blindness, largely governed by genetic components that lead to structural and/or functional perturbations and blindness [1–5]. Photoreceptor and retinal pigment epithelium (RPE) dystrophies are inherited retinal disorders (IRDs) resulting in severe visual impairment that often present during childhood but extend into adulthood [1]. IRDs affect more than two million individuals worldwide and can be organized according to the inheritance pattern (autosomal recessive, autosomal dominant, X-linked or mitochondrial) [4]. Multiple forms of IRDs exist with diverse onset, symptoms, severity, and progression of disease. One well-known IRD is Leber congenital amaurosis (LCA). LCA has the earliest onset and greatest severity of all IRDs with 18 known linked genes (RetNet: https://sph.uth.edu/retnet/disease.html). Retinitis Pigmentosa (RP), another established IRD, shows greater variability in onset and rate of disease progression. Over 40 loci have been identified for non-syndromic RP and 30 genes have been causally related (RetNet: https://sph.uth.edu/retnet/disease.html). RP, also referred to as rod-cone dystrophy, is characterized by an initial loss of rod photoreceptor cells, causing visual field narrowing and night blindness. With no therapy or cure in existence for RP, the disease can eventually progress to cone dysfunction and ultimately total vision loss. Photoreceptor cell death has been widely held as the central event that leads to retinal neurodegeneration and eventually irreversible blindness [1,2]. The apoptotic death of photoreceptor cells is therefore the cornerstone of the pathophysiological process in RP. Recently, many studies have examined the involvement of the endoplasmic reticulum (ER)-unfolded protein response (UPR) in neurodegenerative diseases including Alzheimer disease, Parkinson disease, Huntington disease and Amyotrophic Lateral Sclerosis, all of which have been shown to be associated with heritable mutations that cause the accumulation of misfolded proteins [6–8]. Studies have also provided evidence that misfolded proteins initiate a pathological process that disrupt the ER quality control pathways, leading to ER stress and neuronal cell death [7,8]. The ER is primarily responsible for the proper folding and maturation of secretory and membrane proteins as well as lipid and sterol biosynthesis. Only properly folded proteins can be transported to the Golgi apparatus for further processing; whereas misfolded proteins aggregate in the ER lumen to form insoluble cytotoxic deposits [9,10]. Under severe conditions of ER stress, such as rising levels and prolonged accumulation of misfolded mutant proteins, cells activate the unfolded protein response (UPR) signaling network in an effort to restore protein homeostasis. This novel role of the ER is mediated by three major signal transducers: PKR-like endoplasmic reticulum kinase (PERK), inositol-requiring enzyme 1 (IRE1), and activating transcription factor 6 (ATF6) (Fig 1) [11–14]. The UPR relieves ER stress and restores protein homeostasis through three complementary strategies: (1) suppression of protein translation of misfolded/ unfolded proteins; (2) induction of ER-related molecular chaperones to promote correct folding of the misfolded proteins, and (3) activation of the ER-associated protein degradation (ERAD) system to remove the misfolded proteins [6–8]. The neural sensory retina is a highly specialized part of the central nervous system that requires a well-organized means of maintaining a vast array of proteins required for normal function. This highly regulated protein homeostasis process involves precise control of protein synthesis, protein folding and protein transport and is facilitated by several molecular chaperones [14]. It is therefore not surprising that IRDs caused by mutations in retina-specific genes lead to protein misfolding, aggregation, mislocalization and up-regulation of the ER-UPR in an attempt to restore protein homeostasis [10,15]. Specifically, genetic defects in β-*PDE6*, Clarin1 (*CLRN1*), carbonic anhydrase (*CAIV*), and most notably rhodopsin (*RHO*), have been shown to induce these stress response pathways [16–24]. In these cases, the UPR is unable to restore normal protein homeostasis and apoptosis of photoreceptor cells commence.

Tubby-like protein-1 (TULP1) is a photoreceptor-specific protein that is involved in the transport of several phototransduction proteins synthesized in the inner segment and destined for the outer segment (IS and OS, respectively) [25–27]. TULP1 is localized to the IS, the cellular compartment housing the ER, Golgi apparatus and other biosynthetic machinery, and has been proposed to function as a chaperone or adaptor protein linking cargo-laden vesicles with motor proteins for transport [25–27]. Mutations in the *TULP1* gene have been shown to be the underlying cause of an early-onset, severe form of autosomal recessive RP (arRP) and LCA [28–30]. However, the molecular mechanisms by which mutant TULP1 leads to photoreceptor cell death have not been identified. To the best of our knowledge, we are the first to describe the mechanism of photoreceptor cell death in disease-associated *TULP1* missense mutations. We investigated this issue using *in-vitro* and *in-vivo* models and report that missense mutations express as misfolded protein products that accumulate within the ER. Although the ER-UPR stress complex is initially able to manage misfolded TULP1 protein, its continued presence eventually leads to cellular apoptosis.

## Materials and Methods

### Materials

All chemicals, unless stated otherwise, were purchased from Sigma-Aldrich (St. Louis, MO, USA).

### *In-silico* Analyses of *TULP1* Mutations

Protein stability of four missense *TULP1* mutations were evaluated using the programs SIFT (http://sift.jcvi.org/), PolyPhen 2.0 (http://genetics.bwh.harvard.edu/pph2/), and I-Mutant 3.0 (http://gpcr.biocomp.unibo.it/cgi/predictors/I-Mutant3.0/I-Mutant3.0.cgi) [31–34]. SIFT predicts whether an amino acid substitution affects protein function. Mutations with a SIFT score of < -2.5 are predicted to be pathogenic. PolyPhen 2.0 predicts the damaging effects of missense mutations on protein structure and function using physical and comparative considerations. A mutation is qualitatively appraised, as benign, possibly damaging, or probably damaging based on pairs of false positive rate (FPR) thresholds. I-Mutant 3.0 predicts the thermostability changes created by a single point mutation on a native protein sequence. Values of < -0.5 predict decreased protein stability with the potential for aggregation.

### TULP1 Structure Analysis

Atomic coordinates for human TULP1 were obtained from the Protein Data Bank under accession code 3C5N (unpublished). A dimer is present in the asymmetric unit but a single monomer was considered for the structural analysis. Amino acid substitutions (missense mutations) at D94, R420, I459 and F491 were generated and visualized using the program COOT [35]. Structural figures were generated with PyMOL (http://www.schrodinger.com/pymol/).

### Animals

Basic animal maintenance included conventional micro-isolator caging, and food was available ad lib. Animals were kept in a 12-hour light–dark cycle with an ambient light intensity at the eye level of the mice of 85 ± 18 lux. In addition to these details, the Biological Resource Unit of the Cleveland Clinic follows the Guide for the Care and Use of Laboratory Animals. No deviations occurred. WT C57BL/6 mice were purchased from Jackson Laboratory (Bar Harbor, Maine). All experiments on animals were approved by the Institutional Animal Care and Use Committee (IACUC) of the Cleveland Clinic and were performed in compliance with the ARVO Statement for the Use of Animals in Ophthalmic and Vision Research. Animals were monitored daily during the duration of the project by animal care personnel and veterinary technicians. Animals subjected to sub-retinal injection and electroporation were monitored by members of the Hagstrom laboratory until fully recovered and returned to mother. For mice P5 or older, CO_2_ asphyxiation followed by cervical dislocation was used. For P0-P5 mice, CO_2_ asphyxiation followed by decapitation was performed. Buprenorphine was given once prior to sub-retinal injection. Proparacaine was give once prior to sub-retinal injection. No adverse reaction was seen. For each TULP1 construct, 10 mixed male and female mice retinas were injected per construct (WT-TULP1 n = 10, F491L-TULP1 n = 10, and D94Y n = 10). 3 retinas were utilized for co-localization studies and 7 retinas were pooled for quantitative real-time PCR (qRT-PCR) analysis.

### Human TULP1 Plasmid Construction

The full-length human TULP1 (Gene ID: ENST00000229771) open-reading frame was amplified from human retina cDNA by PCR using gene specific primers TULP1-Fwd (5′-GGAAGATCTCATGCCTCTGCGGATGAA-3′) with TULP1-Rev (5′-GTAGAATTCGCTCGCAAGCCAGCTTCCC-3′) and proof reading AccuPrime Pfx enzyme (Invitrogen, Carlsbad, CA). The full-length human TULP1 cDNA fragment was subsequently cloned in-frame into the mammalian expression vector pEGFP-N1 (Clontech, Mountain View, CA) containing a GFP tag (WT-TULP1). The WT-TULP1 plasmid was used as a template to engineer each of the human *TULP1* gene mutations (D94Y-TULP1, R420P-TULP1, I459K-TULP1, and F491L-TULP1) by *in-vitro* site directed mutagenesis (Quick Change II XL: Stratagene/ Agilent, Santa Clara, CA) as previously described [36,37]. Appropriate construction of the WT-TULP1 and mutant TULP1 plasmids were verified by DNA sequence analysis of both strands using pEGFP-N1 vector primers.

### Cell Culture, Transient Transfection and Confocal Imaging

The human retinal pigment epithelial cell line (hTERT-RPE-1: ATCC CRL 4000) or human embryonic kidney cells (HEK293T: ATCC CRL 3216) were obtained from American Type Culture Collection (ATCC, Manassas, VA) and cultured at 37°C with 5% CO_2_ in F12:DMEM or DMEM medium (Life Technologies, Carlsbad, CA) respectively, both containing high-glucose supplemented with 10% fetal bovine serum (FBS) and 1% penicillin-streptomycin sulfate. For subcellular localization assays, hTERT-RPE-1 cells were grown on glass coverslips in 6-well plates (Life Technologies, Carlsbad, CA). For western blot analysis, hTERT-RPE-1 cells were cultured in 100 cm^2^ dishes. At 60% confluence, cells were transfected with 3 μg of purified plasmid DNA (WT-TULP1, D94Y-TULP1, R420P-TULP1, I459K-TULP1, and F491L-TULP1) using FuGENE HD or XtremeGENE HD (Roche, Indianapolis, IN) as previously described [36,38]. Fourteen days after transfection, hTERT-RPE-1 cells were washed twice with 1X PBS and fixed with cold methanol: acetone fixative (50:50 v/v) for 15 min at -20°C. Visualization of the ER in fixed cells was achieved by staining cells with anti-Calnexin antibodies (an ER resident protein) (Cell Signaling Technologies, Carlsbad, CA). Subcellular localization patterns of the GFP-fused TULP1 proteins and the ER was achieved by imaging the cells at 488 nm (TULP1-GFP) and at 568 nm (ER) wavelengths respectively using a Leica SP8 confocal microscope. Z-stack images were collected using a 63x oil immersion objective. All experiments were carried out in triplicate. Approximately 100 cells from 10–15 fields per experiment were observed for individual TULP1 protein localization patterns.

### Endoplasmic Reticulum Isolation

Isolation of the ER from transfected hTERT-RPE-1 cells was performed with an endoplasmic reticulum isolation kit (Sigma ER0100-1KT) according to the manufacturer’s directions. Approximately 25 μg of the final ER extract was re-suspended in 1X Laemmli sample buffer, electrophoresed on 4–12% SDS-PAGE gels and then transferred to PVDF membranes. Antibodies against ER-proteins Calnexin and calreticulin (Cell Signaling Technologies, Carlsbad, CA) were used to define the ER-containing fractions. Total protein from untransfected hTERT-RPE-1 and HEK293T (25 μg) cell lines served as controls. To identify cross contamination in the ER-fractions, membranes were stripped and probed for mitochondria using anti-COX IV antibodies (Cell Signaling Technologies, Beverly, MA) and for the Golgi using anti-Golgin97 antibodies (Cell Signaling Technologies, Beverly, MA). Anti-GFP antibodies (Life Technologies, Beverly, MA) and anti-Tulp1 antibodies [25] were used to detect localization of GFP-fused recombinant WT and mutant TULP1 proteins.

### Sub-retinal Injections and Electroporation

Approximately 0.5 μg (diluted in 1X PBS containing 0.1% fast green) of either WT-TULP1, D94Y-TULP1, or F491L-TULP1 plasmid was injected into the subretinal space between the retina and RPE of post natal day 1 (P1) mice as described previously [39,40]. D94Y was chosen as the only known N-terminal mutation while F491L was chosen as a common C-terminal mutation found in RP patients [41,42]. For co-localization studies, WT or mutant TULP1 plasmids were co-electroporated with the mCherry-tagged ER-marker Sec61b plasmid DNA (mCherry-Sec61b, a gift from Dr. Gia Voeltz (Addgene #49155)). Only the right eye of each mouse was injected with the left eye being used as an internal control. Immediately after injection, tweezer electrodes were placed on opposing sides of the pup’s head, and electric pulses (5 square pulses of 80V, 50ms shocks, 950ms intervals) were applied using an electroporator (Harvard Apparatus, Holliston, MA). Mice were then revived on a heat pad and developed to P30 for evaluation.

### Retina Dissection and Confocal Microscopy

P30 mouse eyes were enucleated and fixed by immersion in 4% paraformaldehyde in phosphate buffer for 90 min at room temperature (RT). Eyes were incubated in a sucrose gradient of 5% sucrose in phosphate buffer (SPB) for 15 minutes at RT, 15% SPB for 15 minutes at RT, 30% SPB for 1 hour at RT, overnight in a 70:30 v/v ratio of OCT:SPB solution (Tissue-Tek, Sakura Finetech, Torrance, CA) at 4°C. Eyes were then mounted in cyro-moulds containing 70:30 v/v ratio of OCT: SPB solution and frozen on a dry-ice bath containing 100% ethanol. 10-μm thick sections were cut using a cryostat. WT or mutant TULP1 GFP-fused plasmid (WT-TULP1, D94Y-TULP1, and F491L-TULP1) expression was visualized at 488 nm (green fluorescence). The ER marker (mCherry-Sec61b) expression was visualized at 594 nm (red fluorescence). Sections were mounted in Vectashield containing DAPI (Vector Laboratories, Burlingame, CA). Z-stack images were collected using a Leica SP8 confocal microscope (Leica, Germany) and processed with the Leica Viewer software.

### TUNEL Assays

Detection of apoptosis by the TUNEL (TdT mediated dUTP-biotin nick end labeling) method was performed on day 14 post transfection of WT or mutant TULP1 plasmids in hTERT-RPE-1 cells as outlined by the manufacturer (In-situ cell death detection system TMR Red, Roche, Indianapolis, IN). Post TUNEL staining, cells were spread on glass slides with Vectashield containing DAPI (Vector Laboratories, Burlingame, CA). TUNEL-positive cells were visualized immediately and counted using a Leica SP8 confocal microscope (Leica, Germany) with a 20x objective. Approximately 200 cells from five randomly selected fields of cells from each transfection were evaluated. The percentage of TUNEL-positive apoptotic cells was represented relative to the total cells counted.

### Western Blot Analysis

Total protein from untransfected HEK293T cells, untransfected hTERT-RPE-1 cells, or transfected hTERT-RPE-1 cells were isolated using the M-PER protein lysis buffer (ThermoScientific, Beverly, MA) containing protease inhibitors (Roche, Indianapolis, IN). Approximately 25 μg of total protein was electrophoresed on 4–12% SDS-PAGE gels and then transferred to PVDF membranes. Membranes were then probed with primary antibodies against α-actin at 1:10,000 dilution, Tulp1 [25], BiP, phosphorylated PERK (pPERK), CHOP, XBP1 (which detected both the uncleaved XBP1 and cleaved XBP1s protein fragment), calreticulin, Calnexin and Golgi97 all at 1:1000 dilution (Cell Signaling Technology, Beverly, MA) in antibody buffer (0.2% Triton X-100, 2% BSA, 1X PBS). HRP conjugated secondary antibodies (BioRad, Hercules, CA) were used at 1:10,000 dilution.

### Quantitative Real Time-PCR

RNA was obtained from micro-dissection of TULP1 plasmid DNA transfected areas of mouse retina visualized by GFP expression, isolated using Trizol reagent, and processed as described previously [36]. One microgram of total RNA was reverse transcribed using the iScript cDNA Synthesis Kit (BioRad, Hercules, CA). Quantitative Real-Time PCR (qRT-PCR) was carried out using the SYBR green 1 chemistry (EvaGreen SYBR Green Master Mix, Biotium, Hayward, CA) and gene specific primers pairs for human TULP1 (forward 5'-AGAACAAGACGCTGGAGAGC-3' and reverse 5'-GTTGAGGGTGTAGGAGCCAC-3'), mouse CHOP (forward 5’-ATATCTCATCCCCAGGAAACG-3’ and reverse 5’-TCTTCCTTGCTCTTCCTCCTC-3’), mouse XBP1s (forward 5’-ACATCTTCCCATGGACTCTG-3’ and reverse 5’-TAGGTCCTTCTGGGTAGACC-3’), mouse PERK (forward 5’-TTGGCCACTTTGAACTTCGG-3’ and reverse 5’-CGCCATGACCTTCCAATCAG-3’), mouse eIF2α (forward 5’-—CGGTCAAAATTCGAGCAGAT-3’ and reverse 5’-TGATGGGCATGGTTTCTGTA -3’), mouse ATF4 (forward 5’-CGAGATGAGCTTCCTGAACAGC-3’ and reverse 5’-GGAAAAGGCATCCTCCTTGC-3’), mouse BiP (forward 5'- GCTTCGTGTCTCCTCCTGACCCCG-3' and reverse 5'- TAGGAGTCCAGCAACAGGCTGTGGCC-3'), mouse GAPDH (forward 5'-CAGGAGCGAGACCCCACTAACATC-3' and reverse 5'-CGACATACTCAGCACCGGCCTCAC-3'), GFP (forward 5′-CCACATGAAGCAGCAGGACTT-3′) and (reverse 5′-GGTGCGCTCCTGGACGTA-3′), and mouse IRE1 (forward 5’-CTGTGGTCAAGATGGACTGG-3’ and reverse 5’-GAAGCGGGAAGTGAAGTAGC-3’).GAPDH was used as the endogenous control and all values were normalized to TULP1 mRNA expression to control for electroporation and transfection efficiency. Samples for QRT-PCR experiments were assayed in triplicate for each ER-UPR marker using the Applied BioSystems 7500 qRT-PCR machine. Each experiment was repeated twice, using newly synthesized cDNA. The ΔΔCt method was employed to calculate fold changes (Applied Biosystems User Bulletin No. 2 (P/N 4303859).

### Statistical Analyses

Results are presented as mean±s.d. and the number of experiments is indicated in the figure legends. All determinations for each experiment were performed at least in duplicate. Statistical significance was assessed using the two-tailed Student’s *t*-test. For western blot analysis, relative intensities of each band were quantified (densitometry) using the ImageJ Software version 1.49 and normalized to the loading control Actin. qRT-PCR analysis was normalized to GAPDH (as a mRNA quantity control) and GFP (as a transfection efficiency control). The ΔΔCt method was employed to calculate fold changes. This was calculated by first averaging the cycle threshold (Ct) values from the housekeeping gene (GAPDH) and the genes being tested in the experimental and control conditions. Statistical significance was assessed by using the two-tailed Student’s *t*-test.

## Results

### *In-silico* Analyses Predict Mutant TULP1 Proteins to be Misfolded and likely Pathogenic

To date, approximately 40 disease-causing *TULP1* mutations have been identified (Human Gene Mutation Database). To investigate whether *TULP1* mutations produce misfolded proteins, we focused on missense mutations that either: 1) affect the highly conserved C-terminal tubby domain with the highest prevalence in RP or LCA patients (R420P, I450K, and F491L) or 2) were uniquely located outside the tubby domain (D94Y) [41,42].

Protein stability of four missense *TULP1* mutations (D94Y, R420P, I459K, and F491L) was first evaluated using three bioinformatic programs (SIFT, PolyPhen 2.0 and I-Mutant 3.0). All algorithms predicted that these four *TULP1* missense mutations would have deleterious effects on the resultant protein, with the mutant proteins being unstable under physiological conditions and therefore pathological (Table 1). Next, we used the protein structure program RaptorX to predict whether the secondary and tertiary structure of mutant TULP1 proteins would be structurally altered compared to WT TULP1. This structural analysis predicted that all four mutant TULP1 proteins would have an abnormal open loop structure. Alterations to the tertiary structure of TULP1, caused by these missense mutations, are predicted to result in an unstable protein with the potential for aggregation and therefore likely pathological (Table 1 and S1 Fig).

**Table 1:** *In-silico* analysis of *TULP1* mutations on protein stability and folding.

*TULP1* Mutation	SIFT Score: Prediction	PolyPhen 2.0: Protein Structure Prediction	I-Mutant 3.0: Protein Thermostability
D94Y	-2.121: neutral	Possibly damaging	-0.05; likely unstable
R420P	-4.436: pathogenic	Probably damaging	-0.94; unstable
I459K	-6.027: pathogenic	Probably damaging	-1.80; unstable
F491L	-5.576: pathogenic	Probably damaging	-1.55; unstable

The availability of the TULP1 C-terminal tubby domain (residues 243 through 505) crystal structure allowed us to further evaluate the location of the C-terminal *TULP1* mutations and their effects on native folding and structure (S2A Fig). The locations of Arg420, Ile459 and Phe491 are shown as orange transparent spheres. All mutations are located on one side of the β-barrel within or adjacent to three consecutive beta strands (S8-S10). Arg420 lies between a mobile loop and the N-terminal end of β-strand S8 of the β-barrel motif. Substitution of a proline residue at this position (R420P) could introduce an abnormal kink that disrupts protein folding (S2B Fig). Unstructured regions within protein chains like the loop in which Arg420 resides are frequently of functional importance [43,44]. Thus, in the fraction of the mutant protein that may be properly folded, it is possible that the proline substitution could also shift the conformational distribution of the loop to a less functional ensemble. Introduction of a positively charged lysine residue at amino acid position 459 (I459K) into the apolar environment in which the isoleucine side chain resides would be energetically unfavorable and likely result in reduced protein stability or improper folding (S2C Fig). In a similar fashion, a leucine substitution at amino acid position 491 (F491L) would disrupt packing between the β-strand and the central α-helix within the β-barrel motif (S2D Fig). In summary, *in-silico* analysis of all four *TULP1* mutations predict that each resultant protein would be misfolded and/or unstable with altered protein structures that, when expressed, would likely be pathogenic. Further *in-vitro* experiments are required to determine precise protein stability and degradation rates of mutant TULP1 compared to WT-TULP1 proteins.

### Mutant TULP1 Proteins Localize to the Endoplasmic Reticulum *In-vitro*

**Fig 2:**
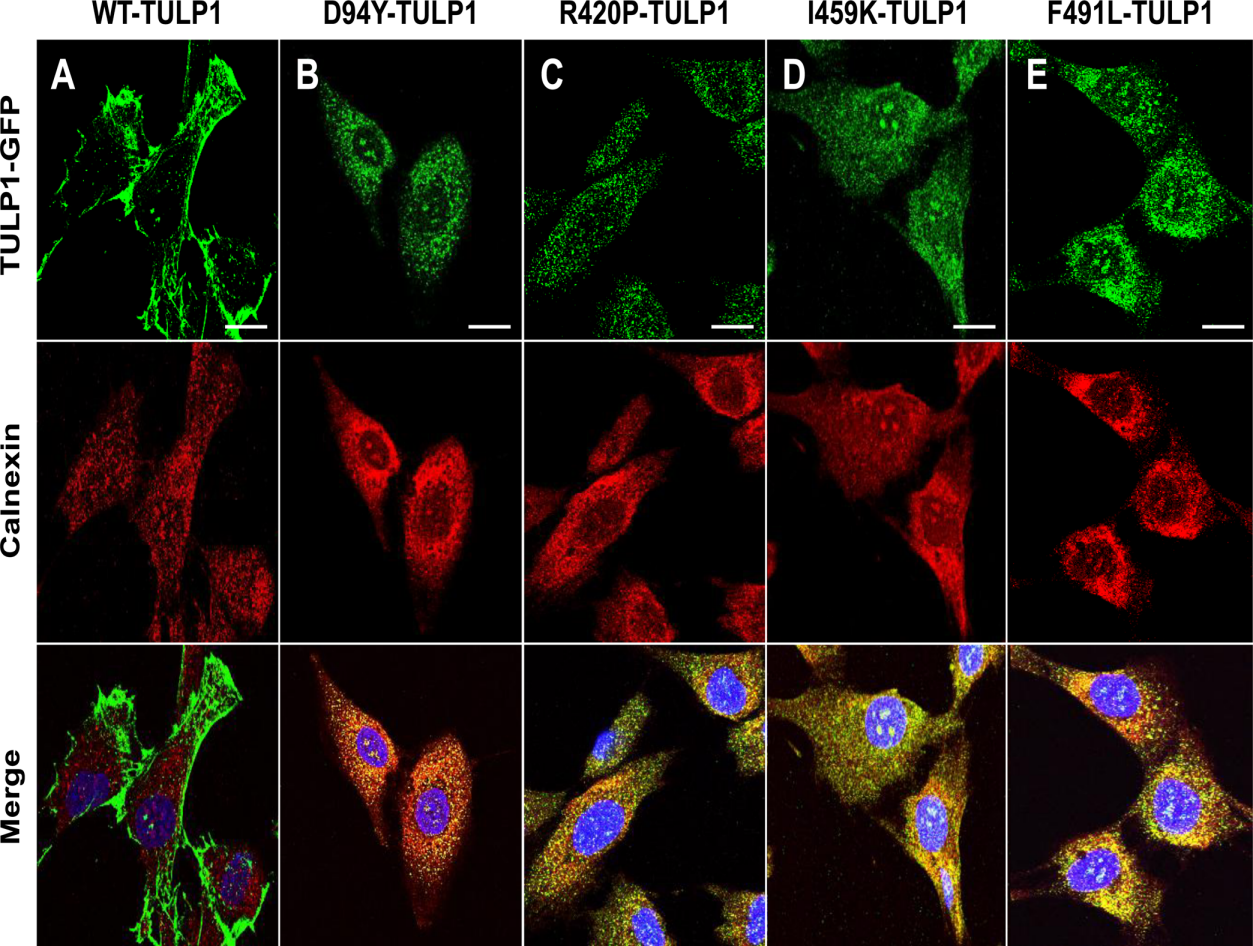
Co-localization of mutant TULP1 protein with the ER-resident protein Calnexin. Immunofluorescent localization of GFP-fused TULP1 (green) and Calnexin (red) in WT and mutant TULP1 expressing cells. Nuclei were stained using DAPI (blue) (A) WT-TULP1 expressing cells displayed predominant plasma membrane and some cytoplasmic staining patterns. (B-E) In contrast all four mutant TULP1 expressing cell lines (D94Y, R420P, I459K and F491L) displayed punctate co-localization patterns with the ER-resident marker Calnexin (merge: yellow). Scale bar = 10 μM. Approximately 100 cells per transfection were counted. Experiments were repeated twice.

To confirm *in-silico* predictions and determine the subcellular distribution patterns of mutant TULP1 we initiated experiments to determine if mutant TULP1 is indeed mistrafficked. We transiently expressed the GFP-fused WT and mutant TULP1 (D94Y, R420P, I459K, and F491L) plasmids in hTERT-RPE-1 cells. Using immunostaining and confocal microscopy, we compared the localization patterns of resultant GFP-fused proteins. In Fig 2A, WT-TULP1 was distributed predominantly to the plasma membrane and processes of the cells, similar to that previously observed in COS7 cells [45]. In contrast, each mutant TULP1 protein showed punctate staining within the cytoplasm of hTERT-RPE-1 cells in a pattern resembling that of the ER. When we immunostained the cells with antibodies against the ER-resident protein calnexin, the merged images showed that the four mutant TULP1 proteins, but not WT-TULP1, co-localized with calnexin, indicating that the mutant TULP1 proteins were located in the ER (Fig 2B–2E).

**Fig 3:**
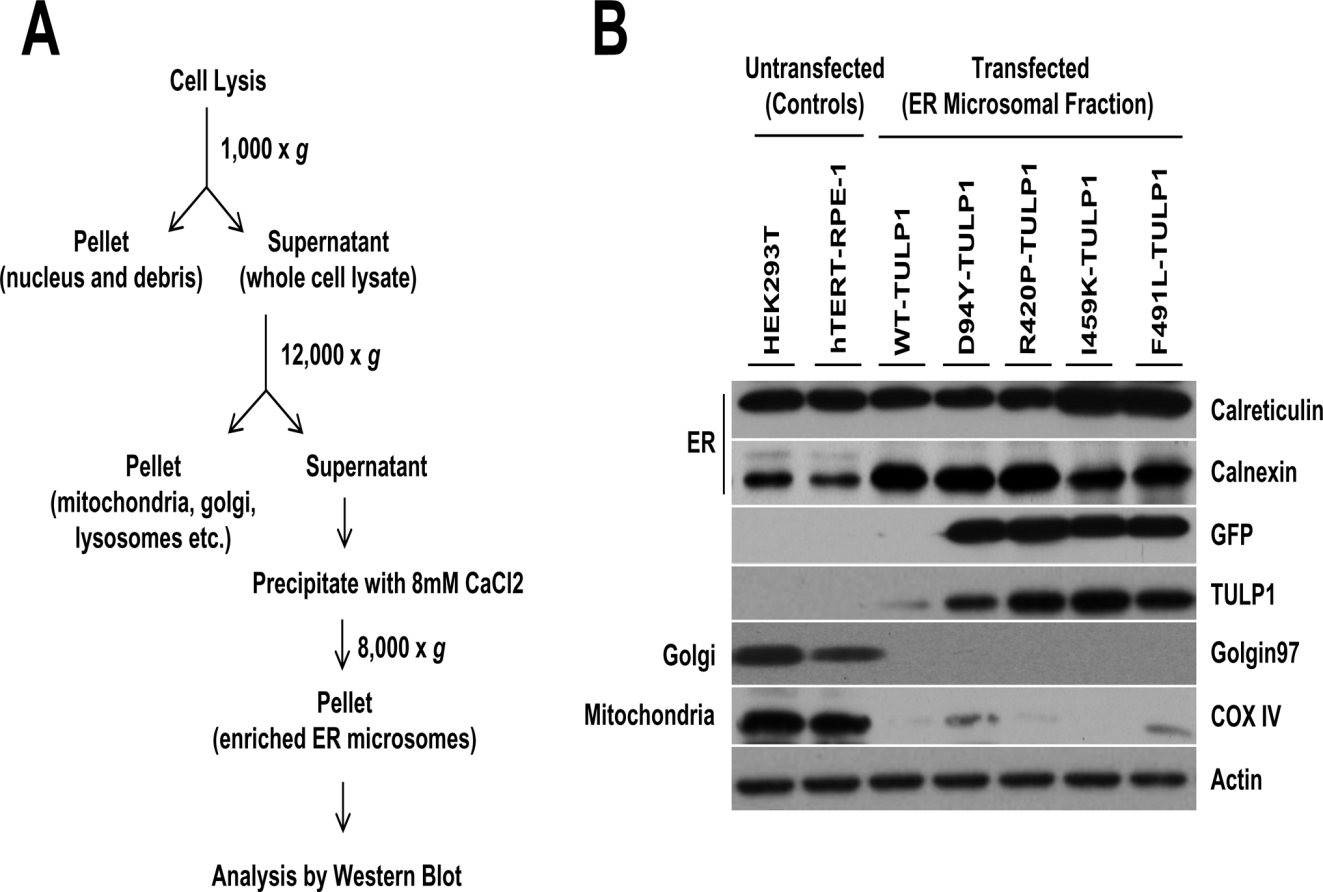
Retention of mutant TULP1 in the ER of fractionated cells. (A) Overview of the subcellular fractionation procedure used for the isolation of ER microsomes. (B) Western blot analysis of ER microsomes isolated from GFP-fused WT or mutant TULP1 expressing hTERT-RPE-1 cells. Antibodies against Calnexin and Calreticulin were used to determine the ER fractions; whereas antibodies against Golgin97 and COX IV were used to determine the presence of Golgi or mitochondria, respectively. Expression of mutant TULP1 proteins (D94Y, R420P, I459K and F491L) was retained within the ER. Total cell lysate from untransfected HEK293T or hTERT-RPE-1 cells were used as controls. Actin was used a protein loading control.

To confirm our immunofluorescence results, we isolated ER microsomes from hTERT-RPE-1 cells expressing mutant TULP1 using a subcellular organelle isolation protocol (Fig 3A). ER fractions of untransfected and TULP1-expressing cells showed a strong presence of the ER-resident proteins, calreticulin and calnexin, indicating successful isolation of ER microsomes (Fig 3B). To estimate purity of isolated ER fractions, we immunoblotted for COX IV and Golgin97, markers of the mitochondria and Golgi respectively. This analysis indicated minimal mitochondrial, but no Golgi, presence in the ER microsomal fractions (Fig 3B). Immunostaining for the presence of GFP-fused TULP1 proteins, using both anti-GFP and anti-Tulp1 antibodies, showed that all four mutant TULP1 proteins but not WT-TULP1 were expressed within the ER microsomal fractions (Fig 3B). Therefore, by immunofluorescence and subcellular organelle fractionation approaches, we confirmed that recombinant mutant TULP1 proteins are expressed and retained within the ER fractions of hTERT-RPE-1 cells.

### Mutant TULP1 Proteins cause Induction of the ER-UPR Stress Complex *In-vitro*

**Fig 4:**
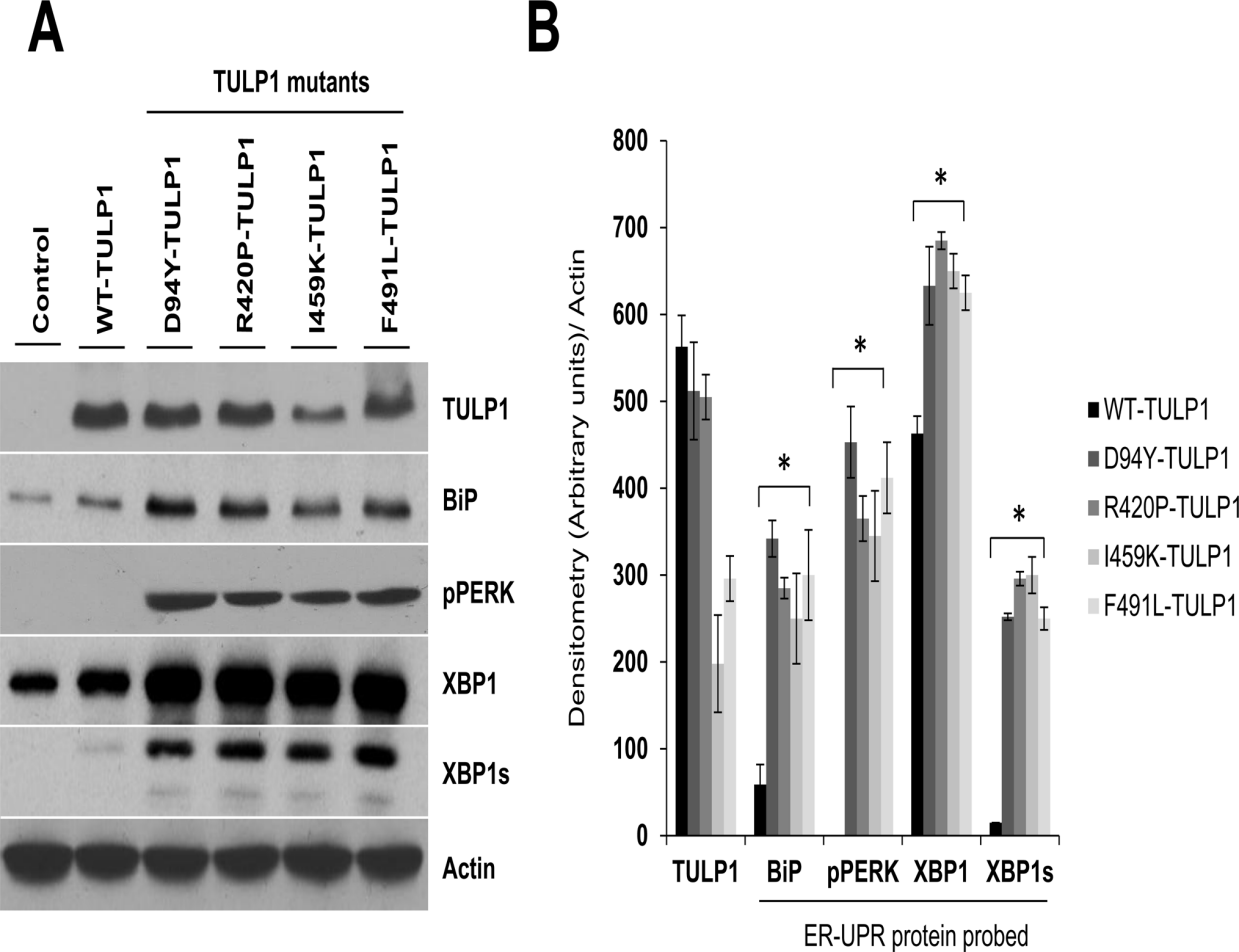
Induction of the ER-UPR complex in cells expressing mutant TULP1. (A) Western blot analysis using antibodies against BiP, phosphorylated PERK (pPERK), XBP1 and XBP1s, showed induction of UPR stress proteins in cells expressing mutant forms of TULP1. Actin was used a protein loading control. Western blot experiments were repeated twice. (B) Quantification of ER-stress protein markers was performed using densitometry in mutant TULP1 expressing cells compared to WT-TULP1 expressing cells. The relative band intensity of each protein was normalized to Actin. Data is presented as arbitrary units from two separate western blot densitometry analyses. Statistical significance was calculated using the two-tailed Student’s *t*-test. * = p< 0.001. Since the untransfected or WT-TULP1 expressing cells revealed no activation of pPERK protein, the pPERK densitometry value was arbitrary set to 1 (arbitrary units/ pixel intensity) for statistical calculations.

The accumulation of misfolded proteins within the ER causes cellular stress as evidenced by an increase in expression of ER chaperones, such as BiP, and proteins signaling activation of the unfolded protein response (UPR) complex [46,47]. To determine if accumulation of misfolded mutant TULP1 proteins activate the ER-UPR complex, we examined markers corresponding to the two major apoptotic arms of the UPR pathways, PERK and IRE1. hTERT-RPE-1 cell lines transfected with WT or mutant TULP1 all expressed TULP1 protein (Fig 4A). Those expressing mutant TULP1 have significantly elevated levels of the ER chaperone protein, BiP, as compared to untransfected (control) or WT-TULP1 expressing cells (Fig 4A). Activation of BiP leads to its dissociation from PERK and IRE1, the two ER signaling transducers, with subsequent oligomerization and autophosporylation of these proteins, ultimately resulting in activation of their corresponding downstream apoptotic pathways [48]. To test for this, we first probed for activation of PERK by measuring phosphorylated PERK (pPERK) protein levels in these cells. While we observed a strong induction of pPERK protein levels in the four mutant TULP1-expressing cells, the untransfected or WT-TULP1 expressing cells revealed no activation of this protein (Fig 4A). We then tested for activation of IRE1 by examining protein levels of its downstream target, XBP1. Activation of IRE1 leads to splicing of *XBP1* messenger RNA (mRNA), producing a potent transcription factor named XBP1s. XBP1s then enters the nucleus and activates the transcription of chaperone-encoding genes that leads to downstream signaling of apoptotic events [10–14]. While we observed endogenous levels of un-spliced XBP1 in all cell lines, only cells expressing mutant TULP1 protein showed the presence of XBP1s (Fig 4A). To evaluate statistical significance of ER-UPR protein induction in mutant vs WT-TULP1 expressing cells, the relative intensities of each ER-UPR protein band were quantified by densitometry and normalized to the loading control Actin. This analysis showed a statistically significant induction of all ER-UPR proteins in mutant TULP1 expressing cells as compared to WT-TULP1 expressing cells (Fig 4B) (p< 0.001). No expression of pPERK was detected in WT-TULP1 expressing cells (Fig 4A). These results indicate that in the presence of mutant TULP1 proteins, expression of BiP is elevated, likely in an effort to promote proper protein folding, followed by upregulation of pPERK and XBP1s. Thus *in-vitro*, accumulation of misfolded mutant TULP1 proteins in the ER can induce the UPR complex likely leading to activation of downstream signaling events.

### Prolonged Retention of Mutant TULP1 *In-vitro* causes Apoptosis

**Fig 5:**
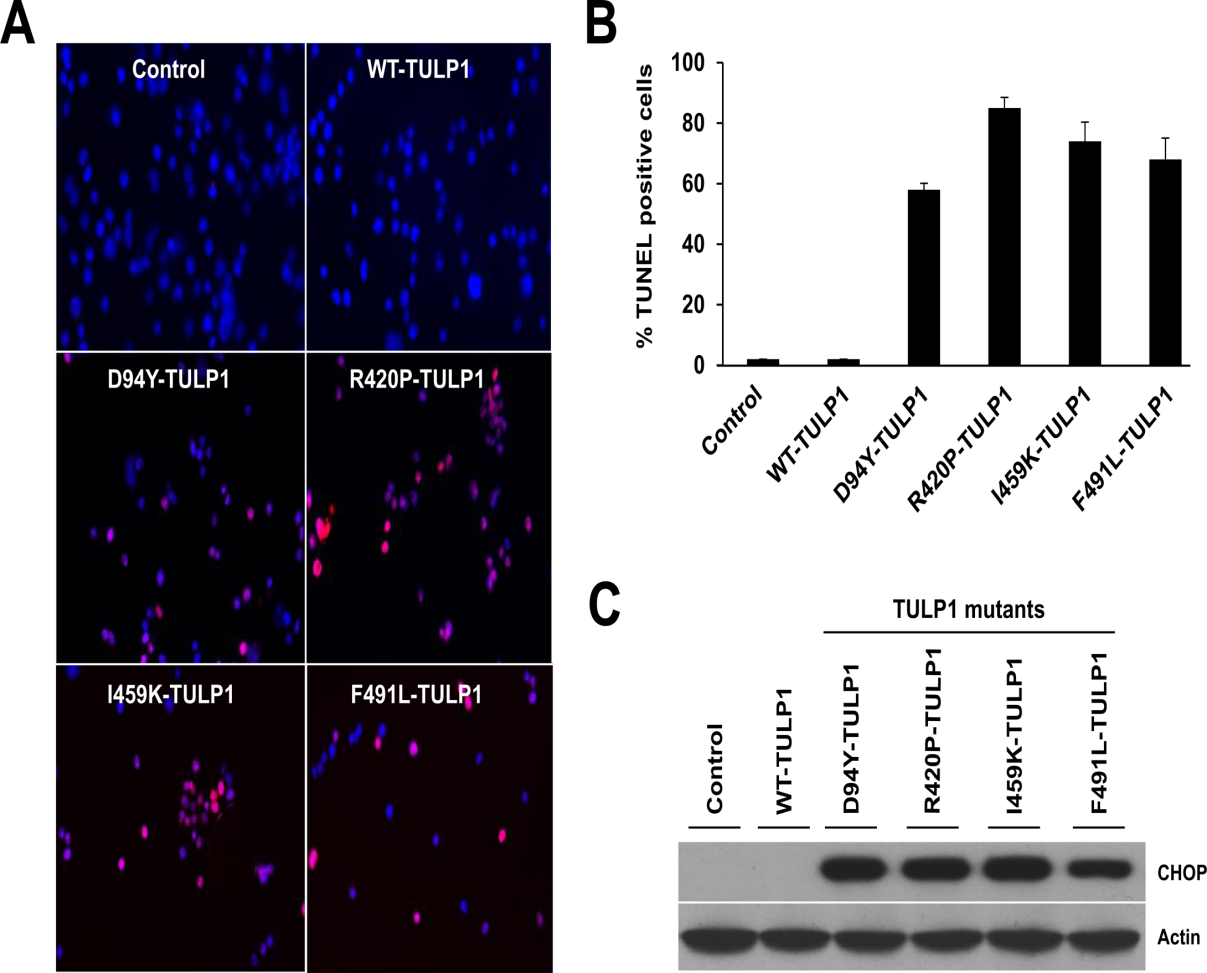
Mutant TULP1 expressing cells undergo apoptosis. (A) Confocal images of untransfected, WT and mutant TULP1 expressing cells stained with TUNEL (red). (B) Quantification of TUNEL-positive nuclei showed statistically significant differences between untransfected, WT and mutant TULP1 expressing cells (p<0.0001). (C) Western blot analysis using antibodies against the pro-apoptotic transcription factor CHOP protein indicate significant induction of CHOP in mutant TULP1 expressing cell lines as compared to WT-TULP1. Approximately 200 cells from five randomly selected fields of cells from each transfection were evaluated.

Prolonged retention of misfolded mutant proteins within the ER can cause apoptotic cell death via the UPR downstream signaling complex [12]. Therefore, we wished to determine if long-term expression of mutant TULP1 proteins in cells induces apoptosis. To achieve this, the recombinant WT and the four mutant TULP1 plasmids (D94Y, R420P, I459K, and F491L) were individually transfected into hTERT-RPE-1 cells. At day 14 post-transfection, cells were collected and subjected to TUNEL-TMR red assay. We quantified the number of nuclei that were TUNEL-positive counting approximately 200 cells per plasmid (Fig 5A and 5B). In contrast to untransfected control and WT-TULP1 transfected cells, which showed <1% TUNEL-positive nuclei, the four mutant TULP1-expressing cell lines showed a significantly higher percentage of TUNEL-positive nuclei (55–85%; R420P > I459K > F491L > D94Y) (Fig 5A and 5B). To identify the possible mechanism of UPR mediated cell death, we measured protein levels of C/EBP homologous protein (CHOP), a downstream pro-apoptotic target of pPERK and IRE1. *CHOP* encodes a transcription factor that promotes cellular apoptosis in response to uncontrolled ER stress [12,49]. Our western blot analysis showed high levels of CHOP expression in all four mutant TULP1-expressing cell lines, while no CHOP protein expression was detected in control or WT-TULP1 expressing cells (Fig 5C). Cumulatively, our results indicate that long term retention of mutant TULP1 within cells can activate the ER-UPR stress complex, leading to the initiation of apoptosis via the CHOP signaling pathways.

### Mutant TULP1 Proteins Localize to the Endoplasmic Reticulum *In-vivo*

To study the molecular mechanism of photoreceptor degeneration in missense *TULP1* mutations, we established an animal model transiently expressing mutant TULP1 protein in photoreceptors. We chose to evaluate the N-terminal D94Y mutation compared to the C-terminal F491L mutation. GFP-fused WT and mutant TULP1 plasmids were transiently expressed in mouse retinas at post natal day 1 (P1) by subretinal injection followed by electroporation [39,40]. An mCherry-tagged ER reporter plasmid (mCh-Sec61b) was co-electroporated with individual TULP1 constructs to visualize the ER. All retinas were examined at P30. At this age, reliable transfection efficiency was seen, with approximately 15–20% of the retina transfected across two separate injection sites (S3A Fig).

**Fig 6:**
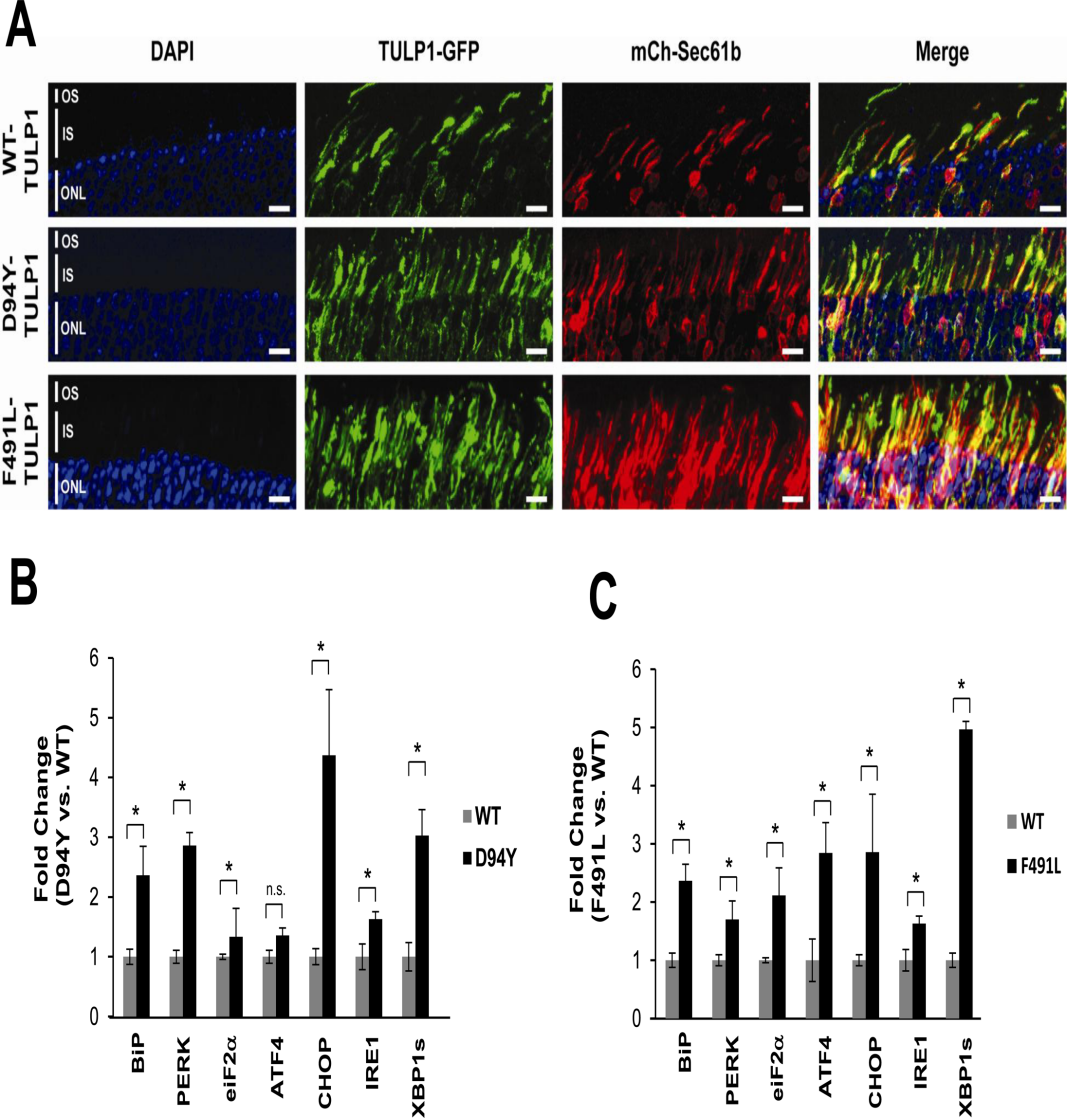
Co-localization and induction of UPR markers in mutant TULP1 expressing photoreceptors. P1 WT mice retinas were co-transfected with GFP-fused (green) plasmids and a mCherry tagged ER reporter (mCh-Sec61b: red). Retinas were harvested and sectioned at P30 (A) Confocal images of retinas show co-localization (merge: yellow) of GFP-fused mutant D94Y-TULP1 and F491L-TULP1 with mCh-Sec61b, the ER resident protein, in the inner segments. Scale bar = 10μM. (B-C) Activation of the ER-UPR stress markers in transgenic mice expressing mutant TULP1 protein. qRT-PCR indicates a 1.3–5 fold increase of multiple ER-UPR stress markers in mutant D94Y-TULP1 (B) or mutant F491L-TULP1 (C) retinas vs WT-TULP1 injected retinas. qRT-PCR analysis was normalized to GAPDH (as a mRNA quantity control) and GFP (as a transfection efficiency control). The ΔΔCt method was employed to calculate fold changes. Samples were assayed in triplicates for each ER-UPR marker and qRT-PCR experiments were repeated twice. Statistical significance was assessed by using the two-tailed Student’s *t*-test. * = p<0.001; n.s. not statistically significant.

Recombinant WT-TULP1 expression matched that of endogenous Tulp1, localizing to the photoreceptor ISs and perikarya [25,26] (Fig 6A). The pEGFP-N1 plasmid backbone (empty vector control) was expressed throughout all layers of the retina, as previously observed [39,40] (S3B Fig). In contrast, mutant TULP1 (D94Y and F491L) expression showed increased aggregation within the IS which co-localized with the ER reporter construct expression (Fig 6A, merged images). These *in-vivo* observations are consistent with our *in-vitro* studies suggesting that mutant TULP1 is retained in the ER.

### Mutant TULP1 Protein causes Induction of the ER-UPR Stress Complex *In-vivo*

After establishing ER localization of mutant TULP1 proteins, we evaluated ER-UPR involvement in our transgenic mice. We used qRT-PCR to determine that retinas expressing the D94Y or F491L TULP1 showed elevated expression of BiP (~2.3 fold), PERK (~1.8 to 2.9 fold), eIF2α (~1.3 to 2.9 fold), ATF4 (~1.3 to 2.0 fold), IRE1 (~1.5 fold) and XBP1s (~3–5 fold) as compared to WT-TULP1 injected eyes (Fig 6B and 6C). The D94Y-TULP1 mutant showed significant statistical differences (p<0.001) in all ER-UPR stress marker mRNA expression levels, with the exception of ATF4 (Fig 6B). The F491L-TULP1 mutant revealed statistically significant differences (p<0.001) in all ER-UPR stress marker mRNA expression levels examined (Fig 6C). This data suggests that both the PERK and IRE1 branches of the ER-UPR are activated in mutant TULP1-expressing retinas. We further probed for induction of the apoptosis precursor, CHOP, and observed a ~2.5 to 4.1 fold increase in mRNA expression in both of the retinas expressing mutant TULP1 relative to the WT-TULP1 retina (Fig 6B and 6C). Thus, we validated our *in-vitro* findings and demonstrated involvement of the two key pro-apoptotic arms of the ER-UPR in mutant TULP1-expressing retinas.

## Discussion

Mutations in the *TULP1* gene have been shown to be the underlying cause of early-onset autosomal recessive retinitis pigmentosa (MIM #600132, RP14) and Leber congenital amaurosis (MIM#613843, LCA15). At the present date, over 40 different *TULP1* mutations have been reported in the Human Gene Mutation Database with most causing missense mutations in the conserved C-terminal tubby domain. Regardless of the type of mutation, clinical presentation remains similar with peripheral vision loss, nyctalopia, and incurable blindness–all secondary to photoreceptor degeneration [50]. No current therapies exist for *TULP1*-associated retinal disease as the pathologic mechanism of photoreceptor degeneration, linking genotype to phenotype, is currently unknown. By modeling the human *TULP1* missense mutations both *in-vitro* and *in-vivo*, our results implicate the ER-UPR as a plausible mechanism for photoreceptor degeneration and for future therapeutic intervention.

Multiple studies have implicated the ER-UPR signaling cascade in other inherited retinal degeneration models, most notably in autosomal dominant RP (adRP) rhodopsin mutations (T17M, P23H, S334ter, rhodopsin-1) [18,51–55]. The ER-UPR through its three main downstream targets–IRE1, PERK, and ATF6 –manages and mitigates the effects of misfolded proteins through chaperone-facilitated proper folding or proteasome-mediated degradation (Fig 1). The pathway initiates with the presence of misfolded proteins. Disassociation of BiP from or direct interaction with misfolded proteins causes IRE1, PERK, and ATF6 activation. IRE1, PERK, and ATF6 respond by balancing the cellular reaction to misfolded proteins between pro-survival or pro-apoptotic. With persistent presence, aberrant proteins overwhelm the ER and ultimately cause cell-induced apoptosis. In light of its relationship to other IRDs, the ER-UPR was a rationale mechanism for photoreceptor cell death caused by mutant TULP1 proteins.

Unsurprisingly, the ER-UPR mechanism is a heavily integrated and dynamic process. Dissection of the relative involvement of each arm of the ER-UPR in cellular fate relays the importance of each UPR product. This is demonstrated in recent studies which have shown that selective activation of IRE1 and ATF6 but not PERK reduced the amounts of misfolded protein in adRP models [51,56]. However, ubiquitous activation of the ER-UPR has been seen in the majority of adRP with increased levels of BiP, IRE1, ATF6, PERK and their respective downstream targets. In addition, CHOP or caspase activation is commonly the finite precursor to apoptosis in these models [18,50].

Our study remains consistent with other IRD models; we demonstrate that missense mutations in *TULP1*-associated arRP modeled *in-vitro* and *in-vivo* cause BiP, IRE1 and PERK induction (Figs 4–6). Specifically, we target IRE1 and PERK because of their well-documented link to apoptosis and photoreceptor degeneration. IRE1 binds pro-apoptotic Bcl2 family members Bax and Bak with tumor necrosis factor receptor-associated factor 2 (TRAF2). This complex activates Jun amino-terminal kinase (JNK) and induces cytochrome c-mediated apoptosis [57]. Separately, CHOP, the downstream target of all three branches of the ER-UPR, is primarily induced by ATF4, the downstream target of PERK. Once CHOP is activated, the cell is expected to undergo apoptosis through multiple mechanisms, such as the suppression of pro-survival Bcl2 [58]. It is theorized that activation of both CHOP and IRE1 mediated apoptotic cascades cumulatively favor cell death [58]. Our study demonstrates activation of the two implicated apoptotic pathways with verification by TUNEL staining *in-vitro*.

Rhodopsin mislocalization could be a possible secondary cause for photoreceptor degeneration in our model. TULP1 is important in the trafficking of proteins to the outer segment of photoreceptor cells; in the absence of TULP1, rhodopsin is retained within the outer nuclear layer and inner segments of photoreceptors [25]. Similar to Class I and II rhodopsin mutations, retention of rhodopsin can cause aggregation within the inner segments that are directly or indirectly cytotoxic to the organelles by creating oxidative species, activating the UPR complex, inhibiting normal cellular function, or requiring unmaintainable metabolism [59]. Although rhodopsin mislocalization may participate in the dysfunction and demise of photoreceptors, our data still supports the retention of misfolded TULP1 protein in the ER and activation of the UPR. However, further evaluation of an animal model that is transgenic for TULP1 and rhodopsin mutations are necessary to parse out the extent or presence of rhodopsin-induced photoreceptor degeneration.

Another rationale that may support degeneration secondary to mislocalization of other proteins relates to the subcellular expression of TULP1. Tulp1 is expected to be a cytosolic protein, yet we see misfolded Tulp1 co-localize to the ER, where the ER-UPR is theorized to function within the lumen. How Tulp1 enters the ER remains unknown; however, Tulp1’s presence in the ER may be required for proper trafficking of or complex formation with its known interacting partners involved in phototransduction (i.e. rhodopsin), structural integrity (i.e. actin), and motor dynamics (i.e. dynamin) [25,27,45,60–63]. Akin to α-synuclein in neurons, absence of properly functioning Tulp1 could disrupt bulk ER to Golgi trafficking, ultimately causing cellular destabilization and proteotoxic stress [64]. Further investigation is required to elucidate whether a lack of Tulp1 or Tulp1 itself is inducing the ER stress seen in the data presented.

Few studies have implicated the ER-UPR with arRP. Rd1 mice, associated with mutations in the β-subunit of the rod photoreceptor-specific cGMP phosphodiesterase 6 gene *(Pde6-β)*, have shown activation of BiP, PERK, and caspase 12 [18]. Separately, *in-vitro* expression of missense mutations of CLRN1, a gene associated with Usher syndrome, is retained within the ER [24]. To our knowledge, our study is unique as it is one of the only arRP models that demonstrate UPR activation with verified apoptosis by TUNEL assay. Our *in-vivo* data reveals a phenotypic activation of the UPR in a transgenic mouse despite the presence of WT protein (Fig 6). Furthermore, as compared to the WT TULP1 mice, expression of N- and C-terminal mutant TULP1 proteins showed elevated BiP, IRE1, PERK, and their respective downstream targets. These findings suggest that mutant TULP1 does in fact cause ER stress and activation of the UPR. A less likely explanation is a dominant-negative effect of the mutant TULP1 as apoptosis is not seen, which is discussed later.

A separate targeted goal of our study was to identify whether missense mutations in the divergent N-terminus created a differential phenotype as compared to the conserved C-terminus, with the hope of elucidating the functionality of TULP1. Clinically, our knowledge of phenotypic differences between N- and C-terminus mutations is limited, as patients often present late with varying manifestation of disease. Our *in-silico* results suggest that missense mutations limited to the tubby domain had decreased protein stability, producing a more deleterious product. In contradistinction, our *in-vitro* and *in-vivo* findings show no difference between mutations regarding the degree of localization within the ER, amount of ER-UPR complex activation, or extent of apoptosis induced. Although *in-silico* results are based on algorithmic prediction, our results could be confounded by the resolution of our study models. More specifically, we employed an over-expression model that could minimize the pathological differences in amino acid structure suggested by our *in-silico* findings. Regardless, our results still establish the ER-UPR as a mechanism of photoreceptor degeneration, but further investigation is required to resolve phenotypic variances.

*In-vivo* we established the ability to express TULP1 protein in the photoreceptor cell, co-localize the mutant TULP1 protein within the ER, and show activation of the ER-UPR stress complex by qRT-PCR (Fig 6B and 6C). However, when probing for apoptosis by the TUNEL assay or through ONL thickness analysis, no evidence of cell death was seen in retinas expressing either the WT or mutant TULP1 proteins (data not shown). A likely explanation for these findings is two-fold: the ability of the ER-UPR to overcome the mutant TULP1 compounded by compensation and redundancy of endogenous mouse Tulp1. In normal ER physiology, the primary function of the ER-UPR stress complex is to manage misfolded proteins by increasing chaperone expression or degrading misfolded protein by an ER-associated protein degradation (ERAD) complex. Once an overwhelming exposure to mutant protein compromises the cell’s functionality, activation of the pro-apoptotic cascade occurs which is thought to be mediated by CHOP. Despite our findings that suggest over-expression of mutant TULP1 induces ER-UPR stress with elevated levels of CHOP, apoptosis did not occur. Reports have suggested that CHOP may not necessarily induce apoptosis, albeit a more likely explanation is that redundant endogenous mouse Tulp1 offsets the deleterious effects of the misfolded protein and prevents mutant TULP1-mediated ER decompensation and apoptosis. Our findings, specifically *in-vitro*, strongly suggest ER-UPR dependent photoreceptor degeneration occurs with mutant TULP1 but warrants further investigation with a complete knock-in model for each mutation. This is particularly necessary as our *in-vivo* model attempts to reconcile a homozygous autosomal recessive mutation. We are currently pursuing this type of animal model using the CRISPR/Cas9 gene-editing technology to recapitulate patients’ gene dosage and spatiotemporal retinal degeneration [65,66]. Of note, correlation of retinal degeneration and activation of the ER-UPR complex has been reported in the *Tubby* mice [67]. Similar to our report, Cai *et*. *al*. also observed induction of key ER-UPR stress markers in the retinas of the *Tubby* mice. Their data also demonstrated that ER stress triggers apoptosis via down-regulation of Bcl2, up-regulation of CHOP (similar to our report) and the activation of NF-кB signaling [67].

In summary, our findings suggest that the ER-UPR is the mechanism by which photoreceptor degeneration occurs in missense mutations of *TULP1*. By identifying the ER-UPR stress complex as a causative pathway for apoptosis, our investigation links known protein intermediates that can be targeted. With a stable cell line or knock-in mouse model, we can utilize and optimize known modulators of the ER-UPR cascade to delay or prevent the development of photoreceptor degeneration [68]. Although clinical trials have directed their attention to managing misfolded proteins in other systemic diseases, only valproic acid, a pharmacological chaperone for unfolded proteins, has been evaluated in patients with RP [69–71]. The attractiveness of pursuing TULP1 as a model for therapeutic targeting is its exclusivity to the photoreceptor cell and direct genotype-phenotype correlation. By elucidating the mechanism of photoreceptor degeneration in this subpopulation of patients with arRP and LCA, we can identify novel therapeutics with measurable clinical outcomes that can directly impact patients. Ideally, TULP1 could act as a model for marrying disease mechanism and therapy for other recessive forms of IRDs.

## Supporting Information

S1 Fig Prediction of protein structure for mutant TULP1. RaptorX (protein structure prediction server) predicts all mutant TULP1 proteins (D94Y, R420P, I459K and F491L) to have misfolded structural conformation changes compared to WT TULP1.
(TIF)

S2 Fig The crystal structure of the TULP1 C-terminal domain. (A) Overall view of the TULP1 C-terminal structure (PDB accession code 3C5N) showing the location of the residues examined in this study that are affected by pathological *TULP1* mutations. The WT residues are shown as orange sticks and transparent spheres. The dashed line indicates a region of the chain that was unresolved in the structure due to intrinsic flexibility. Lack of a full-length TULP1 crystal structure precluded structural analysis of the N-terminal D94 position of the protein. (B) Location of Arg420 between a mobile loop and the N-terminal end of S8. Substitution of a proline residue at this position could introduce an abnormal kink that disrupts protein folding or dynamics of the intrinsically disordered loop located nearby. (C) Hydrophobic pocket surrounding the side chain of Ile459. Introduction of a positively charged lysine residue into this apolar environment would be energetically unfavorable and likely would reduce protein stability or disrupt folding (D) Hydrophobic pocket surrounding the Phe491 side chain. A leucine substitution at this position would disrupt packing between the β-strand and the α-helix within the beta barrel motif likely leading to improper folding and loss of stability In (B-D) residues within 4.5 A of the residue of interest are shown as sticks and colored according to the type of secondary structure they adopt (light blue -β strands and loops; yellow—α helices).
(TIF)

S3 Fig Expression patterns of injected and electroporated recombinant TULP1 in mouse eyes. (A) Flat mount of GFP-fused mutant F491L-TULP1 expressing retina with two separate sites of injection demonstrates ~15% of the retina was transfected. Scale bar = 500μM. (B) The backbone pEGFP-N1 vector is localized throughout all retinal layers in P30 mice.
(TIF)
