# Supplementary figures and images for: Involvement of Endoplasmic Reticulum Stress in TULP1 Induced Retinal Degeneration
Source: PLoS One. 2016 Mar 17;11(3):e0151806. doi: 10.1371/journal.pone.0151806 (PMC4795779; doi:10.1371/journal.pone.0151806)

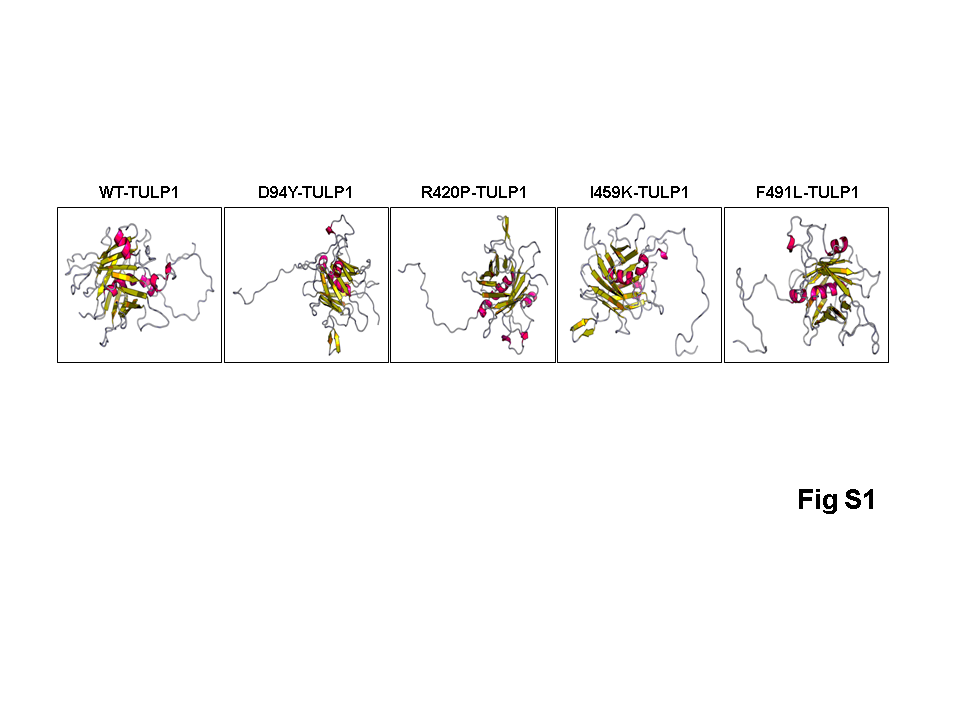

Supplement: S1 Fig — RaptorX (protein structure prediction server) predicts all mutant TULP1 proteins (D94Y, R420P, I459K and F491L) to have misfolded structural conformation changes compared to WT TULP1. (TIF) [file pone.0151806.s001.TIF]

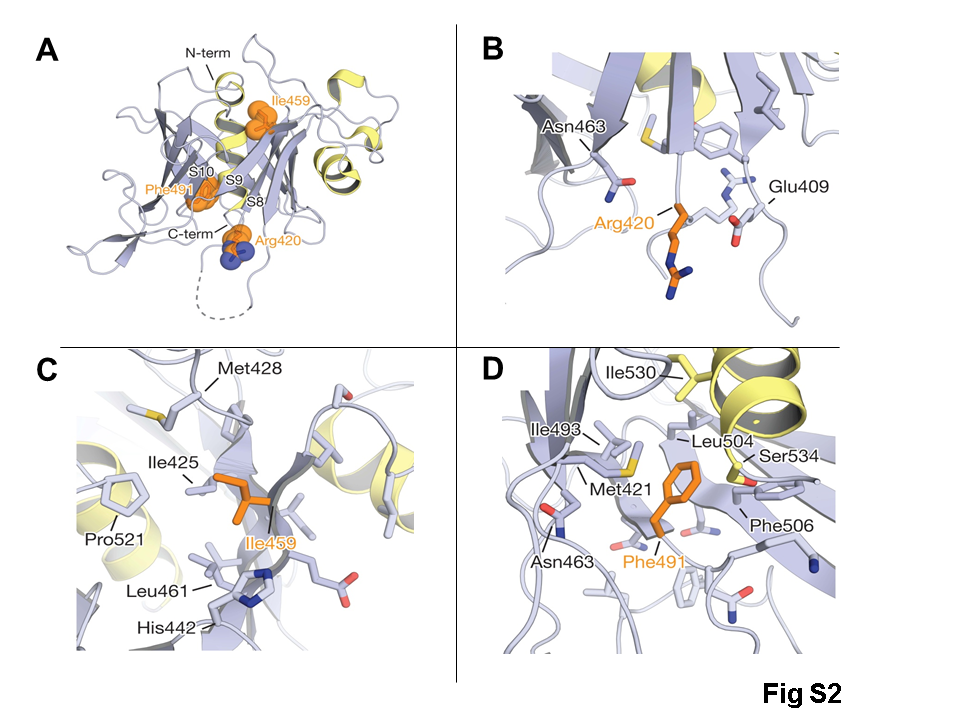

Supplement: S2 Fig — (A) Overall view of the TULP1 C-terminal structure (PDB accession code 3C5N) showing the location of the residues examined in this study that are affected by pathological TULP1 mutations. The WT residues are shown as orange sticks and transparent spheres. The dashed line indicates a region of the chain that was unresolved in the structure due to intrinsic flexibility. Lack of a full-length TULP1 crystal structure precluded structural analysis of the N-terminal D94 position of the protein. (B) Location of Arg420 between a mobile loop and the N-terminal end of S8. Substitution of a proline residue at this position could introduce an abnormal kink that disrupts protein folding or dynamics of the intrinsically disordered loop located nearby. (C) Hydrophobic pocket surrounding the side chain of Ile459. Introduction of a positively charged lysine residue into this apolar environment would be energetically unfavorable and likely would reduce protein stability or disrupt folding (D) Hydrophobic pocket surrounding the Phe491 side chain. A leucine substitution at this position would disrupt packing between the β-strand and the α-helix within the beta barrel motif likely leading to improper folding and loss of stability In (B-D) residues within 4.5 A of the residue of interest are shown as sticks and colored according to the type of secondary structure they adopt (light blue -β strands and loops; yellow—α helices). (TIF) [file pone.0151806.s002.TIF]

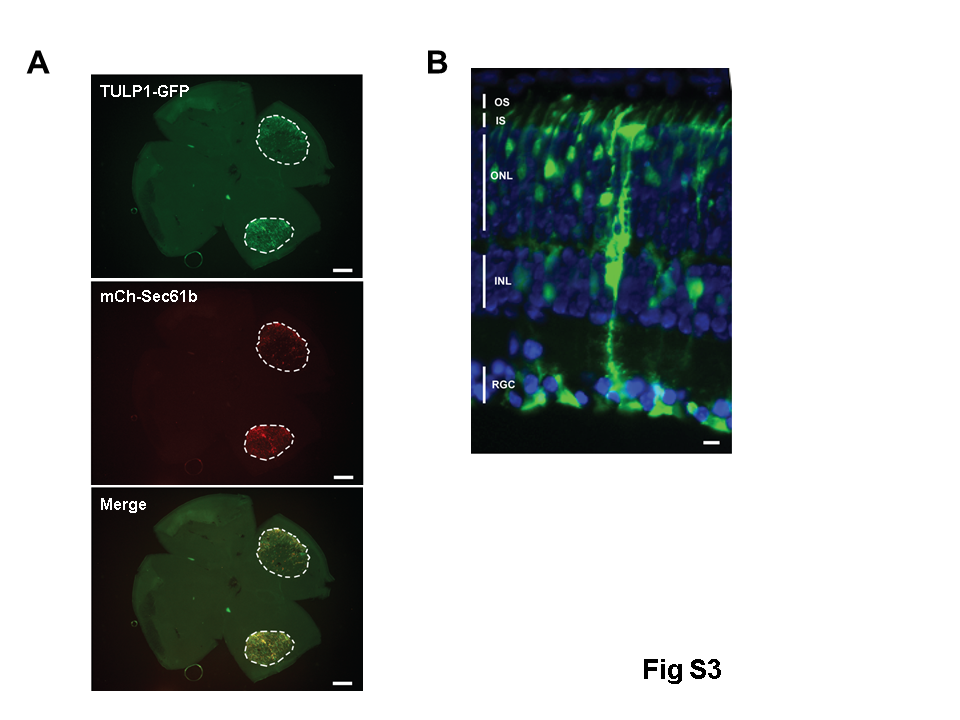

Supplement: S3 Fig — (A) Flat mount of GFP-fused mutant F491L-TULP1 expressing retina with two separate sites of injection demonstrates ~15% of the retina was transfected. Scale bar = 500μM. (B) The backbone pEGFP-N1 vector is localized throughout all retinal layers in P30 mice. (TIF) [file pone.0151806.s003.TIF]
